# A multinational case−control study comparing forensic and non-forensic patients with schizophrenia spectrum disorders: the EU-VIORMED project

**DOI:** 10.1017/S0033291721003433

**Published:** 2023-04

**Authors:** Giovanni de Girolamo, Laura Iozzino, Clarissa Ferrari, Pawel Gosek, Janusz Heitzman, Hans Joachim Salize, Johannes Wancata, Marco Picchioni, Ambra Macis

**Affiliations:** 1 Unit of Epidemiological Psychiatry and Evaluation, IRCCS Istituto Centro San Giovanni di Dio Fatebenefratelli, Brescia, Italy; 2 Unit of Statistics, IRCCS Istituto Centro San Giovanni di Dio Fatebenefratelli, Brescia, Italy; 3 Department of Forensic Psychiatry, Institute of Psychiatry and Neurology, Warsaw, Poland; 4 Central Institute of Mental Health Mannheim, Medical Faculty Mannheim/Heidelberg University, Mannheim, Germany; 5 Clinical Division of Social Psychiatry, Medical University of Vienna, Vienna, Austria; 6 Department of Forensic and Neurodevelopmental Science, Institute of Psychiatry, Psychology and Neuroscience, King's College London, London, UK; 7 St Magnus Hospital, Haslemere, Surrey, UK

**Keywords:** Forensic mental health services, mental disorders, violence

## Abstract

**Background:**

The relationship between schizophrenia and violence is complex. The aim of this multicentre case–control study was to examine and compare the characteristics of a group of forensic psychiatric patients with a schizophrenia spectrum disorders and a history of significant interpersonal violence to a group of patients with the same diagnosis but no lifetime history of interpersonal violence.

**Method:**

Overall, 398 patients (221 forensic and 177 non-forensic patients) were recruited across five European Countries (Italy, Germany, Poland, Austria and the United Kingdom) and assessed using a multidimensional standardised process.

**Results:**

The most common primary diagnosis in both groups was schizophrenia (76.4%), but forensic patients more often met criteria for a comorbid personality disorder, almost always antisocial personality disorder (49.1 *v.* 0%). The forensic patients reported lower levels of disability and better social functioning. Forensic patients were more likely to have been exposed to severe violence in childhood. Education was a protective factor against future violence as well as higher levels of disability, lower social functioning and poorer performances in cognitive processing speed tasks, perhaps as proxy markers of the negative syndrome of schizophrenia. Forensic patients were typically already known to services and in treatment at the time of their index offence, but often poorly compliant.

**Conclusions:**

This study highlights the need for general services to stratify patients under their care for established violence risk factors, to monitor patients for poor compliance and to intervene promptly in order to prevent severe violent incidents in the most clinically vulnerable.

An extensive body of research explores the links between mental disorders and interpersonal violence. The relationship is complex. For example, exposure to violence in childhood is associated with increased rates of later life substance misuse, depression and anxiety (Cerdá, Digangi, Galea, & Koenen, 2012), while people with severe mental disorders are more likely to be the victims of violent crime than other people (Teplin, McClelland, Abram, & Weiner, 2005). Equally people living with mental disorders are more likely to behave violently.

Among the severe mental disorders, there is good evidence to support an independent association between the risk of violence and schizophrenia (Fazel, Gulati, Linsell, Geddes, & Grann, 2009; Walsh, Buchanan, & Fahy, 2002), alcohol and drug dependence (Coid et al., 2006), antisocial personality disorder (Swinson, Webb, & Shaw, 2021; Yu, Geddes, & Fazel, 2012) and psychopathy (Coid & Yang, 2011). Focusing on schizophrenia, the relationship with violence is not strongly linked to the presence of positive psychotic symptoms like delusions or hallucinations (Nolan et al., 2003; Swanson et al., 2008). While certain types of delusions or hallucinatory content may increase the risk of violence in some individuals, they alone do not seem to drive the risk at a group level that patients will be violent (Appelbaum, Robbins, & Monahan, 2000).

One emerging model suggests that amongst patients with schizophrenia who are violent there may be at least two distinct conceptual pathways to violence. One is associated with premorbid conditions linked to violence that include antisocial conduct, a history of previous violence and traumatic experiences, while the other is more intimately linked to the core psychopathology of schizophrenia that the patient experiences (Bo, Abu-Akel, Kongerslev, Haahr, & Simonsen, 2011; Citrome & Volavka, 2014; Hodgins, Piatosa, & Schiffer, 2014; Swanson et al., 2008; Volavka, 2014), possibly strongly modified by the emotional impact of those symptoms at the critical time of the violence (Ullrich, Keers, & Coid, 2014). Childhood trauma is important in a number of ways. People who have experienced childhood adversity, be that physical, sexual or other forms of mistreatment, are at a heightened risk of being violent themselves later in life (Day et al., 2013; Misiak et al., 2017). Being exposed to violence in early childhood is a specific risk factor for conduct disorder (Burke, Loeber, & Birmaher, 2002; Kersten et al., 2017) and is associated with an increased risk of developing psychosis (Larsson et al., 2013; Misiak et al., 2017; Varese et al., 2012), and with a further increased risk of being violent (Bosqui et al., 2014; Macinnes, Macpherson, Austin, & Schwannauer, 2016; Witt, van Dorn, & Fazel, 2013). Finally, a recent systematic review (Green, Browne, & Chou, 2019) found that patients with psychosis and a history of childhood trauma were approximately twice as likely to be violent compared to patients with the same diagnosis, but without trauma experiences.

There is little clear evidence of a link between neuropsychological deficits and the risk of violence in schizophrenia. Any risk may be strongest for executive functions deficits and violence (Bulgari et al., 2017; Hancock, Tapscott, & Hoaken, 2010; Meijers, Harte, Meynen, & Cuijpers, 2017; O'Reilly et al., 2015; Reinharth, Reynolds, Dill, & Serper, 2014). Violence in some is related to frontal and prefrontal cortex dysfunction (Morgan & Lilienfeld, 2000) and thus with deficits in executive functions (Hancock et al., 2010), although the data supporting this relationship is complicated and inconsistent. It may be being influenced by other factors such as age and gender (Dack, Ross, Papadopoulos, Stewart, & Bowers, 2013) and substance misuse (Bowers et al., 2009). It may be that any link with frontal-lobe dysfunction and executive function deficits is strongest in people with schizophrenia or antisocial personality disorders since violent patients perform worse in executive functions (Barkataki et al., 2005; Macinnes et al., 2016).

In order to help us to better understand the relationship between these putative risk factors for violence in schizophrenia we tested their impact as part of the multi-centre ‘*European Study on Risk Factors for Violence in Mental Disorder and Forensic Care-* EU-VIORMED’ (de Girolamo et al., 2019). One aim was to investigate risk factors for violence in schizophrenia spectrum disorders (SSDs).

In line with the existing literature, we expected to find differences between violent and non-violent patients with SSDs in cognitive functioning, personality disorders, alcohol and substance use and their exposure to childhood adversity. We hypothesised that cases would: (i) have a higher prevalence of lifetime history of alcohol and substance use, (ii) have been more often exposed to childhood adversity, and (iii) have worse scores in executive functions compared to the non-violent comparison group.

## Methods

### Participants

EU-VIORMED is a European multicentre observational study. The field work was conducted in five European countries: Austria, Germany, Italy, Poland and the United Kingdom. All subjects were between 18 and 65 years of age with a primary DSM-5 diagnosis made by the treating clinicians of SSD (APA, 2013).

‘Cases’ were patients with a primary diagnosis of a SSD and a history of significant interpersonal violence. They were recruited from multiple forensic institutions in each country (Supplementary file 1S). Significant interpersonal violence was defined as having committed a homicide, attempted homicide or other assaults that caused serious physical injury to another person. ‘Controls’ were gender and age-matched patients with SSDs who have never committed such an act of violence and were recruited from general psychiatric services.

Exclusion criteria included: (i) a confirmed intellectual disability; (ii) a traumatic brain injury or organic brain disorders; (iii) not being able to speak the national language fluently; and (iv) planned discharge from psychiatric or forensic services in the next month.

### Recruitment

In each study centre, treating clinicians invited participants under their care to enter the study. Participants were provided with written information about the study and had an opportunity to ask questions. Informed consent was also sought to allow to collect information from caregivers, family members or case-managers/clinical staff for additional/missing information.

The recruitment of forensic cases was the priority, with care given to matching criteria (e.g. age categories, gender and diagnosis). This helped in finding matched controls, which were recruited from local adult psychiatric services.

Initial plans were to recruit 200 cases and 200 gender- and age-matched controls. However, the worldwide coronavirus outbreak and the resulting restrictions from February 2020 caused recruitment to temporarily halt in every country. The degree and impact of the restrictions varied between the five countries and particularly affected control recruitment. Once recruitment restarted but as some restrictions remained it proved more feasible to over-recruit forensic cases rather than controls.

The study was approved by the Research Ethics Committee (EC) for the coordinating Centre (IRCCS Centro San Giovanni di Dio Fatebenefratelli, Brescia, Italy: n. 74–2018), and by the relevant Research Ethics Committees for each of the participating sites (see at the end of the paper for ECs details). All participants provided written informed consent before entering the study after a full verbal and written description of the study's aims and methods.

### Socio-demographic, clinical, functional and violence assessment

All subjects were evaluated by research assistants employed by the study and centrally trained on each instrument. Socio-demographic, core clinical and criminological and violence risk data were collected using a study-specific Patient Information Form (PIF), an Index Violence Sheet (IVS) and a Risk Factors Questionnaire (RFQ) based on patient interview later cross referenced with the medical and criminal records and clinician review. DSM-5 diagnoses were based on treating clinicians' evaluations extracted from the medical records.

Current psychotic symptoms were assessed using the Positive and Negative Syndrome Scale-PANSS (Kay, Fiszbein, & Opler, 1987), based on a semi-structured patient interview and clinical observation. PANSS scoring used the original standard PANSS model (Kay et al., 1987); the PANSS overall total score ranges from 30 to 210. All research assistants underwent official centralised PANSS training in 2018 provided by the PANSS Institute and were certified PANSS raters.

The World Health Organization Disability Assessment Schedule 2.0-WHODAS 2.0 (Ustün et al., 2010) was used to assess day to day functioning across six functional domains, cognition, mobility, self-care, getting along, life activities and participation. Scores were calculated using a simple sum, yielding a total from 0 to 48, with higher scores indicating more severe problems.

### Cognitive assessment

Cognition was tested with the Brief Assessment of Cognition in Schizophrenia-BACS (Keefe et al., 2004). It assesses six cognitive domains: verbal memory and learning, working memory, motor function, verbal fluency, processing speed and executive function (Keefe et al., 2004).

All assessment instruments were available in official and validated translations.

### Statistical analyses

Frequencies and percentages for categorical variables and means and standard deviation for continuous variables were evaluated. Chi-squared or Fisher's exact test were used according to the nature of the data to compare the categorical variables between the groups. The continuous variables distribution was established by histogram plot and normality tests. *t* tests or the non-parametric Mann−Whitney tests were used for the continuous variables.

Finally, logistic regression models were constructed to quantify the association between the two groups (dichotomous dependent variable) and the variables which significantly differed between cases and controls (independent variables). Each model was adjusted for potential confounders (i.e. sex and country). Then, different multiple logistic regression models were performed to identify the best (in terms of predictive performance) factors with highest predictive risk or protective value for violence.

All analyses were carried out by using SPSS software (IBM Corp. Released 2019. IBM SPSS Statistics for Windows, Version 26.0. Armonk, NY: IBM Corp); significance level was set at 0.05.

## Results

### Sociodemographic and clinical characteristics

Of 575 patients who expressed an initial interest in the study, 175 declined to participate (99 cases, 30.9% and 76 controls, 30.0%). ‘Cases’ and ‘controls’ refusals differed significantly between the five countries (*p* = 0.002 and *p* < 0.001, respectively). In particular, case’ refusal rates in Poland were lower than in the other countries, while controls' refusal rate was higher in Germany and in Poland than in the other countries (Online Supplementary Table S2). Unfortunately, we were unable to collect data on refusers given the prohibition of ECs to acquire any information on these subjects.

The final sample included 398 patients with a primary diagnosis of SSDs: 221 cases had a lifetime history of serious interpersonal violence and 177 controls had no such history. The proportion of cases and controls differed among the five countries (*p* = 0.007). The two groups did not differ in age (*p* = 0.291). The majority of research subjects were males (*N* = 336; 84.4%), with a male excess in the cases (*p* = 0.019), thus all subsequent analyses were adjusted for sex. Cases and controls did not differ on ethnicity, marital and occupational status (Table 1). Compared to controls, cases had lower educational achievement (*p* ⩽ 0.001), spent less time engaged in therapeutic activity (*p* = 0.001) at the time of the project, and more often had children (*p* = 0.004).

**Table 1.** Socio-demographic characteristics of forensic patients with SSD and controls

	Forensic group *N* = 221 *N* (%)	Control group *N* = 177 *N* (%)	*p*-value
Country of recruitment			
Austria	50 (22.6)	50 (28.2)	**0.007**
Germany	36 (16.3)	33 (18.6)	
Italy	39 (17.6)	36 (20.3)	
Poland	56 (25.3)	48 (27.1)	
United Kingdom	40 (18.1)	10 (5.6)	
Sex			
Male	195 (88.2)	141 (79.7)	**0.019**
Female	26 (11.8)	36 (20.3)	
Ethnicity			
White	191 (86.4)	165 (93.2)	0.089
Middle Eastern or Arab (incl. all North African nations)	9 (4.1)	5 (2.8)	
Asian	7 (3.2)	2 (1.1)	
Black/African/Central or South American (Hispanic)	13 (5.9)	3 (1.7)	
Don't know/won't say	1 (0.5)	2 (1.1)	
Age			
18–29	50 (22.6)	52 (29.4)	0.291
30–41	93 (42.1)	60 (33.9)	
42–53	45 (20.4)	40 (22.6)	
54–65	33 (14.9)	25 (14.1)	
Marital status			
Married or cohabiting	10 (4.5)	15 (8.5)	0.223
Single	183 (82.8)	144 (81.4)	
Divorced or widowed	28 (12.7)	18 (10.2)	
Children			
Yes	56 (25.3)	24 (13.6)	**0.004**
No	165 (74.7)	153 (86.4)	
Education years, Mean (s.d.)^a^	11.5 (3.3)	12.9 (3.4)	**<0.001**
Highest occupational status^a^			
Never worked/Student/housewife	32 (14.5)	25 (14.3)	0.427
Unskilled worker	114 (51.6)	77 (44.0)	
Skilled worker	64 (29.0)	63 (36.0)	
Professional	11 (5.0)	10 (5.7)	
Social support^a^^,^^b^			
None	38 (17.2)	18 (10.2)	--- #
Family	170 (76.9)	136 (77.3)	
Friends	28 (12.7)	79 (44.9)	
Other patients	23 (10.4)	3 (1.7)	
All	6 (2.7)	3 (1.7)	
Daily time not engaged^a^			
0–3 h	52 (23.7)	68 (38.4)	**0.001**
3–6 h	72 (32.9)	62 (35.0)	
More than 6 h	95 (43.4)	47 (26.6)	

χ^2^ or Fisher's exact test (when *n* < 5 in at least one cell) has been performed for categorical variables; *t* test has been performed for Education years.

a Frequencies and percentages (for categorical variables) and mean and standard deviations (for continuous variables) have been evaluated considering only the valid cases (i.e. all the cases with no missing data).

b It was not possible to perform the comparison between the two groups because this variable is related to a multiple option question. Consequently, the sum of column percentages is not equal to 100%.

Clinical characteristics are shown in Table 2. The most common primary diagnoses in both groups were schizophrenia (76.4%) and schizoaffective disorder (15.8%). Mean age at first contact with psychiatric services differed significantly between the two groups. However, the mean duration of illness was over 13 years in both groups. Cases were more likely to meet criteria for a comorbid personality disorder than controls (*p* < 0.001); particularly antisocial personality disorder (49.1% *v*. 0%). Cases were more likely to be felt to engage passively rather than actively in their current treatment than controls (*p* < 0.001).

**Table 2.** Baseline clinical characteristics of forensic patients with SSD and controls

	Forensic group *N* = 221 *N* (%)	Control group *N* = 177 *N* (%)	*p*-value
Illness duration (years), mean (s.d.)^a^	13.2 (9.6)	13.7 (10.5)	0.635
Age of first contact with DMHs (years), mean (s.d.)^a^	25.0 (9.l)	22.8 (8.1)	**0.013**
Type of SSD diagnosis			
Schizophrenia	174 (78.7)	130 (73.4)	**<0.001**
Schizoaffective disorders	22 (10.0)	41 (23.2)	
Delusional disorder	12 (5.4)	1 (0.6)	
Brief psychotic disorder	1 (0.5)	1 (0.6)	
Schizophreniform disorder	5 (2.3)	1 (0.6)	
Drug-induced psychosis	7 (3.2)	3 (1.7)	
Comorbidity with personality disorders^a^			
No	152 (70.7)	159 (92.4)	**<0.001**
Yes	63 (29.3)	13 (7.6)	
Type of comorbid personality disorders^a^			
Borderline personality disorder	13 (22.8)	2 (22.2)	**0.002**
Antisocial personality disorder	28 (49.1)	0 (0.0)	
Other	16 (28.1)	7 (77.8)	
Lifetime substance use^a^			
Never	50 (22.7)	46 (26.1)	0.432
Yes	170 (77.3)	130 (73.9)	
Lifetime attempted suicide/self-harm behaviours^a^			
Yes	101 (45.9)	68 (39.5)	0.206
No	119 (54.1)	104 (60.5)	
Collaboration skills in the last year^a^			
Actively seeks treatment, willing to collaborate	86 (39.4)	126 (71.2)	**<0.001**
Wants to be helped, but lacks motivation	30 (13.8)	28 (15.8)	
Passively accepts the treatment/intervention	64 (29.4)	16 (9.0)	
Does not show attention or compreh. for treatment efforts	32 (14.7)	5 (2.8)	
Actively refuses the treatment/intervention	6 (2.8)	2 (1.1)	
Medications			
No	3 (1.4)	4 (2.3)	0.705
Yes	218 (98.6)	173 (97.7)	
Antipsychotics^a^			
No	3 (1.4)	6 (3.5)	0.191
Yes	214 (98.6)	165 (96.5)	

χ^2^ or Fisher's exact test (when *n* < 5 in at least one cell) has been performed for categorical variables; *t* test has been performed for illness duration and age of first contact with DMHs.

a Frequencies and percentages (for categorical variables) and mean and standard deviations (for continuous variables) have been evaluated considering only the valid cases (i.e. all the cases with no missing data).

There were no differences between the two groups in lifetime alcohol and substance use problems (*p* = 0.432).

There were no differences in lifetime suicidal and self-harm behaviour (*p* = 0.206).

### Criminological history of the forensic sample

About 50% (*N* = 104) of cases were detained as a consequence of the most severe form of violent crime, homicide or attempted homicide. The index offence was felt to have been premeditated in over a quarter of cases (26.9%). In the vast majority of cases the violent offence was committed while the patient was mentally unwell (96.3%) at the material time. This was generally due to the presence of psychotic symptoms (94.1%) that the majority already exhibited (65.2%). Furthermore, the majority of cases were in contact with psychiatric services (78.1%) and were prescribed antipsychotic medications (88.1%), though 86.0% were non-compliant with their medicines at the time they committed the index violence. A majority (147 out of 221, 66.5%) of cases had a history of previous violence before their index offence. The nature of that previous violence was generally less severe, the majority were lower-level assaults with (61.2%) or without injury (42.2%), verbal aggression (33.3%) and robbery (30.6%) (see online Supplementary Table S3 and S4).

### Psychopathology, psychosocial functioning and cognition

No significant differences were observed on the current PANSS total score between the two groups (*p* = 0.226) (Table 3), however controls had more severe current positive symptoms (mean score: 15.6, s.d. = 5.7 for controls *v*. mean score: 14.8, s.d. = 6.9 for cases; *p* = 0.020).

**Table 3.** Baseline assessment: clinician-administered assessment tools

	Forensic group *N* = 221 Mean (s.d.)	Control group *N* = 177 Mean (s.d.)	*p* value	Cut-off
PANSS		#		
Positive symptoms^a^	14.8 (6.9)	15.6 (5.7)	**0.020**	
Negative symptoms^a^	18.9 (7.7)	18.3 (6.5)	0.789	
General psychopathology^a^	33.9 (11.2)	34.5 (9.2)	0.121	
Total score^a^	67.8 (23.0)	68.5 (18.5)	0.226	
WHODAS 2.0				
Total score^a^	8.0 (8.4)	12.8 (8.0)	**<0.001**	
BACS				
Verbal memory^a^^,^^b^	32.6 (11.5)	35.6 (12.2)	**0.015**	**>33.01**
Working memory^a^^,^^b^	15.6 (4.7)	16.4 (4.8)	0.177	>14.93
Token motor task^a^^,^^b^	57.7 (16.8)	59.3 (17.3)	0.368	>68.77
Verbal fluency^a^^,^^b^	36.6 (12.6)	39.7 (12.9)	**0.021**	**>31.68**
Symbol coding task^a^^,^^b^	35.4 (12.9)	41.6 (14.1)	**<0.001**	**>40.49**
Tower of London^a^^,^^b^	14.4 (5.2)	14.9 (5.1)	0.320	>12.37

PANSS, Positive and Negative Syndrome Scale; WHODAS 2.0, World Health Organization Disability Assessment Schedule 2.0; BACS, Brief Assessment of Cognition in Schizophrenia.

Mann−Whitney non-parametric test has been performed for all PANSS scores, for WHODAS 2.0 overall score, for BACS Digits sequencing task, token motor task and Tower of London; *t* test has been performed for BACS List learning, verbal fluency and symbol coding task.

a Mean and standard deviations have been evaluated considering only valid cases (i.e. all cases with no missing data).

b Raw scores.

Cases functioned better socially on the WHODAS, reflecting lower disability and better functioning (mean score: 8.0, s.d. = 8.4 for cases *v.* mean score: 12.8, s.d. = 8.0 for controls; *p* < 0.001).

Cases generally had lower scores than controls in all subtests of the BACS except for the tower of London, the digits sequencing and the token motor task on which no differences were found.

### History of victimisation and violence

There were no group differences in self-reported exposure to (*p* = 0.337) or having been the victim of familial physical or sexual violence (*p* = 0.186) between cases and controls, as children. However, among those who had been exposed, there were further significant differences in the frequency of violent exposure (more cases reported both frequently witnessing and being the victims of violence than controls, *p* = 0.002 for frequently witnessing and *p* = 0.008 for being victim), and in the severity of these episodes (some relatives got medical attention as a consequence of the violent episode, *p* = 0.013).

Considering violence outside the home, cases more often reported being the victim of violence, being beaten, kicked or punched (*p* = 0.001) as adults (*p* = 0.002) and more often needed medical attention because of violence (*p* = 0.032) than controls (see online Supplementary Table S3).

Online Supplementary Fig. S1 shows the comparative personality disorder and victimology data for the two groups. More cases had a comorbid personality disorder than controls (29.3% *v.* 7.6%). Of cases who had a comorbid personality disorder 49.1% were antisocial, while no controls had this diagnosis. The percentage of cases and controls with comorbid borderline personality disorder was similar between the two groups. Moreover 31.3% of cases and 26.7% of controls had witnessed violence while 37.0% of cases and 30.4% of controls had been the victim of violence.

### Risk factors for violence

Logistic models were then performed to evaluate the strength of association between the significant variables shown in Tables 1–4 and the two groups. Different models were performed for each significant variable, used as the independent variable, while the group variable was entered as the dependent one. Moreover, since the two groups significantly differed for country and sex, all models were adjusted for these two factors to take into account their potential confounding effect. All adjusted odds ratios (ORs) are reported in Online Supplementary Table S5.

**Table 4.** History of victimisation and violence

	Forensic group *N* = 221 *N* (%)	Control group *N* = 177 *N* (%)	*p*-value
Witness of physical and/or sexual violence in the family^a^			
Yes	66 (31.3)	43 (26.7)	0.337
No	145 (68.7)	118 (73.3)	
When witness of violence^a^			
Early childhood – childhood – adolescence (0–18 years)	62 (93.9)	40 (95.2)	1.000
Adulthood	4 (6.1)	2 (4.8)	
Frequency of witness of violence?^a^			
Rarely	14 (22.2)	24 (55.8)	**0.002**
Occasionally	17 (27.0)	8 (18.6)	
Often	32 (50.8)	11 (25.6)	
Medical attention needed after witnessed violence^a^			
Yes	18 (30.0)	4 (9.5)	**0.013**
No	42 (70.0)	38 (90.5)	
Victim of physical and/or sexual violence in the family^a^			
Yes	77 (37.0)	49 (30.4)	0.186
No	131 (63.0)	112 (69.6)	
When victim of violence^a^			
Early childhood – childhood – adolescence (0–18 years)	72 (94.7)	45 (91.8)	1.000
Adulthood	4 (5.3)	4 (8.2)	
Frequency of victim of violence^a^			
Rarely	23 (31.5)	28 (57.1)	**0.008**
Occasionally	24 (32.9)	14 (28.6)	
Often	26 (35.6)	7 (14.3)	
Medical attention needed after violence^a^			
Yes	9 (12.0)	3 (6.4)	0.311
No	66 (88.0)	44 (93.6)	
Beaten, kicked or punched by someone?^a^			
Yes	149 (70.6)	87 (53.7)	**0.001**
No	62 (29.4)	75 (46.3)	
When beaten, kicked or punched^a^			
Early childhood – childhood – adolescence (0–18 years)	83 (56.8)	67 (77.0)	**0.002**
Adulthood	63 (43.2)	20 (23.0)	
Frequency of beaten, kicked or punched^a^			
Rarely	64 (44.8)	53 (60.9)	0.056
Occasionally	47 (32.9)	19 (21.8)	
Often	32 (22.4)	15 (17.2)	
Medical attention needed after having beaten, kicked or punched^a^			
Yes	43 (29.9)	15 (17.2)	**0.032**
No	101 (70.1)	72 (82.8)	

a Frequencies and percentages have been evaluated considering only valid cases (i.e. all cases with no missing data). Chi squared or Fisher's exact test (when *n* < 5 in at least one cell) has been performed.

Finally, different multiple logistic regression models were performed using a backward method for variables selection. These models were used to identify which were the strongest risk and protective factors for violence. Four multiple logistic models were performed for socio-demographic variables, clinical features, clinical assessments and history of victimisation, respectively. All models were adjusted for sex and country of recruitment to address their potential confounding effect. The final results of the backward selection are shown in Table 5. The strongest socio-demographic, clinical and clinical assessment variables are shown, together with their respective ORs. No results are shown for the history of victimisation model because, when considering all these variables together, no significant factors emerged. In general, values of OR greater than 1 indicate a risk factor for violence, while values smaller than 1 point to a protective role against violence. Among socio-demographic variables, factors that indicated a higher probability of belonging to the forensic group were having children, to be male and the amount of time not engaged in any activity. In detail, those with children and male patients were respectively 2.01 and 1.92 times more likely to be a case; similarly, patients who had more than 6 h not engaged were 2.54 times more likely to be in the violent group than those who were not engaged for less than 3 h.

**Table 5.** Results of multiple logistic models: association between the socio-demographic features, clinical variables and clinical assessment tools (independent variables) and the two groups (forensic and control group)

Independent variable (reference category)	Odds ratio (95% CI)	*p*-value
Model 1. Socio-demographic variables		
Children (*No*)	2.01 (1.14; 3.53)	**0.015**
Education years	0.88 (0.82; 0.94)	**<0.001**
Sex (*Female*)	1.92 (1.04; 3.53)	**0.036**
Daily time not engaged (*0–3 h*)		
3–6 h	1.36 (0.79; 2.33)	0.270
⩾6 h	2.54 (1.43; 4.49)	**0.001**
Country (*Austria*)		
Germany	1.84(0.92; 3.68)	0.083
Italy	0.80 (0.42;1.53)	0.496
Poland	1.94 (1.05; 3.58)	**0.034**
United Kingdom	4.64 (1.99;10.85)	**<0.001**
Model 2. Clinical variables		
Type of Comorbid personality disorders (*Other*)		
Borderline personality disorder	2.84 (0.50; 16.10)	0.237
Antisocial personality disorder	ND	ND
Model 3. Assessment scales		
WHODAS 2.0 Total score	0.89 (0.86; 0.92)	**<0.001**
BACS – Symbol coding	0.96 (0.94; 0.98)	**<0001**

WHODAS 2.0, World Health Organization Disability Assessment Schedule 2.0; BACS, Brief Assessment of Cognition in Schizophrenia; ND, not defined.

All models have been adjusted for sex and country. Backward method for variable selection has been used. Only those variables which remained in the last iteration of the backward method have been reported.

Education was a protective factor against violence: each more year in education was linked to a 12% reduction in the probability to being in the violent group. Finally, country was significantly associated with the group variable, due to the higher percentage of cases recruited in Poland and in the United Kingdom.

Among clinical variables, no significant variables emerged from the backward selection. Finally, higher scores on the WHODAS total score and in BACS Symbol coding were associated with a lower probability to be in the forensic group. In particular, a unit increase of these two scales was associated with a decrease of 11 and 4%, respectively, in the probability of belonging to the forensic group.

## Discussion

To our knowledge, EU-VIORMED is the first international study of people with SSDs who have committed a violent offence and were admitted to forensic psychiatry units.

Overall, what emerges and the persistent challenge for services is firstly that there are only very limited clinical differences between patients with SSDs who commit significant acts of violence and those who have not, and secondly most patients who offend violently are in contact with psychiatric services before they offend.

### Psychopathology and psychosocial functioning

Violent cases had very similar total PANSS ratings to patients with no history of violence at the time of assessment, while controls had slightly higher positive symptoms ratings. That data replicated earlier findings that when engaged in treatment, forensic patients often experience lower levels of psychotic symptoms than their counterparts in general adult services. Clearly the methodological problem in this study to test the impact of psychotic symptoms was that the PANSS ratings were made on average 4.5 years after the patients' admission to a forensic hospital and when all of the cases were well established on an antipsychotic. This study design cannot tell us about the impact of psychotic symptoms on the individual at the time of the offense. In the future more detailed assessments of psychopathology at time of the offence or soon after, using structured tools as well as the emotional impact of those symptoms need to be done.

The PANSS mean total score found among cases in this study (67.8 ± 23.0) was very similar to the PANSS mean total score (65.2, CI 95% 57.7–72.7) found in a recent meta-analysis of 27 studies of 1662 forensic inpatients (Buizza et al., under review). The scores are consistent to the patients being ‘mildly ill’ (Nicotra, Casu, Piras, & Marchese, 2015). In a systematic review of 136 RCTs of 11 774 subjects with SSDs which used the PANSS, PANSS mean scores at baseline were 21.4 ± 5.4 for positive, 23.2 ± 6.3 for negative, 42.1 ± 8.9 for general psychopathology and 88.3 ± 16.0 for the total score (Matsusaki, Kaneko, & Narukawa, 2018). In another recent meta-analysis (Fazel, Smith, Chang, & Geddes, 2018) including 47 trials comparing antipsychotic medicines to placebo, the authors again found higher PANSS total scores, ranging from 57.6 to 100.8 with a mean of 92.5 (s.d. = 7.6). This data shows that violent patients across the five EU countries who were able to take part in this study, but were still inpatients in forensic hospitals, were comparatively well treated; the prolonged exposure to antipsychotic treatment leads to lower PANSS ratings as compared to patients enrolled for antipsychotic trials. Similarly, and in line with previous studies, our data show that patients with SSD and a past history of severe violence function at a higher level on the WHODAS than controls.

We found that forensic patients had on average more children than controls; there is interesting existing data that has shown that many people in forensic services do already have children before they enter services (Chao & Kuti, 2009; Gow, Choo, Darjee, Gould, & Steele, 2010). This may seem counterintuitive, but a closer inspection of our data suggests some possible reasons. Violent cases in our sample had better levels of functioning than the non-violent controls. It is possible that forensic services treat a relatively high functioning group of people, but who pose a risk of violence. Adult psychiatric services have now evolved to focus on a group of more functionally disabled group of patients, who as a result are less likely to establish and maintain adult sexual relationships, thus they are less likely to have children. An alternative possibility is that the high rates of antisocial personality disorder in the violent cases also reflects a group of subjects who are less able or prepared to engage in effective family planning.

### Substance use and history of violence

The co-occurrence of a severe mental disorder and active alcohol or substance misuse increases the risk of violent behaviour (Iozzino, Ferrari, Large, Nielssen, & de Girolamo, 2015; Volavka & Swanson, 2010). In our study however the percentages of cases and controls with a history of alcohol and substance use were broadly speaking similar, emphasising the point that despite the well-established association, alcohol and drug use is common in people with SSDs who have never been violent. Furthermore, in this study almost one in five of the cases had not used any alcohol or substances in their lifetime.

It is clear that some people with psychotic disorders can engage in severe violence even in absence of alcohol and substance use (Fazel et al., 2009; Gosek, Kotowska, Rowińska-Garbień, Bartczak, & Heitzman, 2020; Volavka, 2014). Equally alcohol and substance misuse remains one of the main potentially modifiable risk factors for the prevention of violence in people with SSDs (Cavalera et al., 2020).

### Personality disorders and violence

There was a significant difference in the proportion of cases meeting clinical criteria (as assessed by the treating clinicians) for at least one personality disorder, most frequently antisocial personality disorder, compared to controls. Overall, data suggest that rates of personality disorder do genuinely vary according to the psychiatric setting, with forensic settings having more than adult inpatient settings, that have more than adult community settings. Given the links with violence (Duggan & Howard, 2009; Yu et al., 2012), antisocial personality disorder is common in forensic settings.

Two recent prospective studies (Bottesi et al., 2021; Candini et al., 2018) comparing patients with and without a history of violence found that the violent group scored significantly higher on the Millon Clinical Multiaxial Inventory (MCMI-III) for antisocial, sadistic, borderline, and paranoid personality scales. Furthermore, the most significant predictor of aggressive behaviour over time was having a primary diagnosis of personality disorders.

Previous studies have suggested that maladaptive personality traits and substance use may indeed act as mediators of the association between psychotic symptoms and violence (Fazel et al., 2009; Volavka, 2014)

### History of victimisation and violence

Between group differences were found for both having witnessed and being subjected to violence. Patients who were later violent were more likely to have been abused both earlier in life and more harshly. Childhood trauma and insecure attachment styles predict later violence risk in forensic populations (Macinnes et al., 2016) and have been replicated in non-clinical offender samples (Dargis, Newman, & Koenigs, 2016; Duke, Pettingell, McMorris, & Borowsky, 2010; Temple et al., 2018). A more recent study has shown that patients with schizophrenia who have been violent are more likely to have been exposed to trauma in childhood than patients who have not been, while both had higher trauma exposure than controls (Storvestre et al., 2020). On the whole, exposure to a range of childhood adversities represents a risk factor for a variety of psychiatric and negative behavioural consequences in adulthood.

### Cognitive functioning

According to the available literature, we expected to find difficulties in specific cognitive tasks, in particular in those assessing executive functions, in patients with a history of violence (Barkataki et al., 2005; Rasmussen, Levander, & Sletvold, 1995). Indeed, we found different cognitive profiles among cases compared to non-violent subjects; in particular there were significant inter-group differences on the BACS-Verbal Memory and on BACS-Verbal Fluency: cases had lower performances than non-violent subjects, although scores were under the clinical cut-off in both groups. On the other hand, pathological scores on the BACS-Token Motor Task were found in both cases and non-violent subjects, but the two groups showed statistically no significant differences, with cases scoring worse than non-violent patients.

Interestingly, the only task in which cases showed an impaired performance, while non-violent patients had normal scores, was the BACS-Symbol Coding Task, a measure of processing speed ability. Processing speed reflects the speed at which different cognitive operations can be executed (Dickinson, Ramsey, & Gold, 2007). It is also sensitive enough to predict patients' functional outcome (Gold, Goldberg, McNary, Dixon, & Lehman, 2002; Keefe, Poe, Walker, Kang, & Harvey, 2006), it is a vulnerability-related component among relatives (Niendam et al., 2003) of people with SSDs and it may be present prior to illness onset (Glahn, Thompson, & Blangero, 2007). This consideration might have significant clinical implications and guide the interventions tailored for patients at illness onset.

### Limitations

Despite the relatively large and multinational sample, this study cannot be considered as generalisable to the wider forensic psychiatry population. Many potentially eligible patients, approximately 30% in both samples, refused to participate, and in line with good ethical practice, we were unable to collect any data on them. It was impossible to determine whether the refusers differed clinically or in risk relevant characteristics from those who were recruited into the study.

Assessment of the patients' symptoms was based on current interview, rather than an assessment at the time of the relevant violent offence. The personality disorder assessment relied on clinicians' judgment and the clinical notes, and was not based on a structured assessment instrument. Cognitive performance could have been affected by patients' psychotic symptoms at the time of testing and their current treatment. The histories of victimisation and trauma were based on self-reports without third party verification.

Finally, despite the experimental design was planned to be sex-balanced, the distribution of cases and controls was imbalanced for this variable due to the problems in recruitment linked to the coronavirus disease 2019 (COVID-19) pandemic. However, this drawback was considered through a modelling adjustment for sex variable.

## Conclusions

Most forensic patients who offended violently were in treatment at the time of the offense, although they were not compliant. Once in treatment forensic patients responded well and manifest relatively low symptom levels and functioned better. They remained relatively poorly engaged with treatment and wider activities. This highlights the need for services engaged in the prevention of violent offenses in people living with SSD to strengthen their therapeutic alliance, maintain contact and surveillance of patients who disengage or drop of out treatment and the need to ensure high levels of treatment concordance.

The evidence from this cross-sectional study does not allow a complete understanding of the directionality of the associations we found, and we cannot rule out reverse causality for some of these. Prospective cohort studies, possibly with long-term follow-ups, will be necessary to disentangle some of the important questions related to the association between severe mental disorders and the risk of violence.

## Data Availability

The project will fully embrace the open access data policy of H2020 to make data FAIR (Findable, Accessible, Interoperable, and Re-usable), and all data gathered in the framework of the project are stored in a public repository (https://doi.org/10.5281/zenodo.4442372) accessible to all scientists willing to carry out additional analyses.

## Supplementary material

For supplementary material accompanying this paper visit https://doi.org/10.1017/S0033291721003433. click here to view supplementary material
