# Supplementary material for: A multinational case−control study comparing forensic and non-forensic patients with schizophrenia spectrum disorders: the EU-VIORMED project
Source: Psychol Med. 2021 Sep 13;53(5):1814–24. doi: 10.1017/S0033291721003433 (PMC10106295; doi:10.1017/S0033291721003433)
Supplement: Supplementary file 1 [file S0033291721003433sup001.docx]

**Supplementary materials**

**TABLE 1S**

**LIST OF FORENSIC FACILITIES RECRUITING FOR THE EU-VIORMED PROJECT**

| COUNTRY | FACILITY NAME AND LOCATION | NUMBER OF FORENSIC BEDS | OFFICIAL SECURITY LEVEL |
| --- | --- | --- | --- |
| AUSTRIA | Justizanstalt Goellersdorf, Lower Austria, Goellersdorf | 145 | NA |
|  | Klinik für Psychiatrie mit forensischem Schwerpunkt, Upper Austria, Linz | 53 | NA |
| GERMANY | Klinik für Forensische Psychiatrie und Psychotherapie, Zentrum für Psychiatrie Nordbaden, Wiesloch | 258 | NA |
|  | Klinik für Forensische Psychiatrie des Pfalzklinikums, Klingenmünster | 185 | NA |
|  | Klinik für Forensische Psychiatrie und Psychotherapie, Weinsberg | 124 | NA |
| ITALY | REMS, Centro Polifunzionale ex Ospedale Stellini, ULSS 9 ‘Scaligera’, Nogara | 130 | NA |
|  | Sistema Polimodulare REMS, Castiglione delle Stiviere | 20 | NA |
|  | REMS Minerva, ASL Roma 5, Rome | 20 | NA |
|  | REMS-D, Azienda USL Toscana Nord-Ovest, Volterra | 20 | NA |
| POLAND | Institute of Psychiatry and Neurology, Department of Forensic Psychiatry, Warsaw | 60 | Medium |
|  | SP ZOZ Psychiatryc Hospital, Department of Forensic Psychiatry, Toszek, | 40 | Medium |
|  | Psychiatric Hospital, Forensic Department, Starogard Gdański | 40 | Low |
| UNITED KINGDOM | South London and Maudsley NHS Foundation Trust, London | 130 | Low and Medium |
|  | South West London and St. Georges Mental Health NHS Trust, London | 75 | Medium |
|  | St. Andrew’s Healthcare | 240 | Low and Medium |

NA : Not Applicable, because there are no different security levels.

**TABLE 2S**

**FREQUENCY AND PERCENTAGE OF REFUSALS AMONG CASES AND CONTROLS**

**IN FIVE PARTICIPATING COUNTRIES**

|  | **Austria** | **Germany** | **Italy** | **Poland** | **UK** | **p-value** |
| --- | --- | --- | --- | --- | --- | --- |
| **Cases invited to join the study** | 84 | 55 | 55 | 63 | 64 |  |
| **Cases who refused to participate** | 34 (40.5) | 19 (34.5) | 15 (27.3) | 7 (11.1) | 24 (37.5) | **0.002** |
| **Controls invited to join the study** | 57 | 58 | 45 | 80 | 14 |  |
| **Controls who refused to participate** | 4 (7.0) | 25 (43.1) | 7 (15.6) | 36 (45.0) | 4 (28.6) | **<0.001** |

**TABLE 3S**

**CRIMINOLOGICAL AND CLINICAL CHARACTERISTICS OF THE FORENSIC SAMPLE AT THE TIME OF THE INDEX VIOLENCE**

|  | **Forensic group**  **N=221**  **N (%)** |
| --- | --- |
| **Type of crime (index violence)** |  |
| *Homicide* | 64 (29.0) |
| *Attempted homicide* | 40 (18.1) |
| *Assault with injury* | 93 (42.1) |
| *Used a weapon/force* | 13 (5.9) |
| *Other* | 11 (5.0) |
| **Type of index violence *** |  |
| *Reactive/Impulsive* | 152 (73.1) |
| *Premeditated* | 56 (26.9) |
| **Abnormal mental state (during the index violence)*** |  |
| *No* | 8 (3.7) |
| *Yes* | 208 (96.3) |
| **Type of abnormal mental state (during the index violence)*** |  |
| *Psychotic disorder* | 192 (94.1) |
| *Mood disorder* | 10 (4.9) |
| *Other* | 2 (1.0) |
| **Substance use disorders (at the time of the index violence)*** |  |
| *No* | 90 (42.9) |
| *Yes* | 120 (57.1) |
| **Already in contact with MHS (at the time of the index violence)*** |  |
| *No* | 48 (21.9) |
| *Yes* | 171 (78.1) |
| **Diagnosis before the index violence if already in contact with MHS*** |  |
| *No* | 4 (2.4) |
| *Psychotic disorder* | 144 (85.2) |
| *Personality disorder* | 5 (3.0) |
| *Other* | 16 (9.4) |
| **Prescribed medications (at the time of the index violence)*** |  |
| *Antipsychotics* | 126 (88.1) |
| *Antidepressants* | 10 (7.0) |
| *Other* | 7 (4.9) |
| **Pharmacological compliance (at the time of the index violence)*** |  |
| *No* | 123 (86.0) |
| *Yes* | 20 (14.0) |
| **History of conviction or arrest for the index violence: FIRST admission at the time of index violence** |  |
| *No* | 12 (5.4) |
| *Yes (current forensic unit)* | 44 (19.9) |
| *Yes (another forensic unit)* | 39 (17.6) |
| *Yes (prison)* | 126 (57.0) |

*Frequencies and percentages have been evaluated only for valid cases (i.e., all cases with no missing data).

**TABLE 4S**

**INFORMATION ABOUT THE HISTORY OF VIOLENCE**

**AND PREVIOUS PSYCHIATRIC HOSPITALIZATIONS**

|  | **Forensic group**  **N=221**  **N (%)** |
| --- | --- |
| **Previous hospitalization for severe mental illness*** |  |
| *No* | 70 (32.1) |
| *Yes* | 148 (67.9) |
| **History of any other violent behaviour lifetime (in addition to the index offence)*** |  |
| *No* | 70 (32.3) |
| *Yes* | 147 (67.7) |
| **Type of offence committed in addition to the index offence (up to 3 options allowed) #** |  |
| *Robbery* | 45 (30.6) |
| *Kidnapping* | 5 (3.4) |
| *Stalking* | 6 (4.1) |
| *Assault with injury* | 90 (61.2) |
| *Assault without injury* | 62 (42.2) |
| *Verbal violence – threat* | 49 (33.3) |
| *Other* | 35 (23.8) |
| **Influence of alcohol or substances when committed the offence *** |  |
| *No* | 53 (24.0) |
| *Yes* | 82 (37.1) |
| **History of conviction or arrest, if committed a violent behaviour lifetime *** |  |
| *No* | 47 (32.9) |
| *Yes* | 96 (67.1) |
| **Age at first admission in the lifetime in a forensic unit (years)**, Mean (SD)* | 33.0 (10.5) |
| **Number of lifetime admissions in forensic units**, Mean (SD)* | 1.5 (1.3) |
| **Total Time spent in forensic units in the lifetime (months)**, Mean (SD)* | 53.6 (52.4) |
| **Total Time spent in prison in the lifetime (months)**, Mean (SD)* | 19.8 (48.3) |

*Frequencies and percentages (for categorical variables) and mean and standard deviations (for continuous variables) have been evaluated only for valid cases (i.e., all cases with no missing data).

# This variable is related to a multiple option question: consequently, the sum of column percentages is not equal to 100%:

**TABLE 5S**

**RESULTS OF LOGISTIC MODELS. ASSOCIATION BETWEEN THE SOCIO-DEMOGRAPHIC AND CLINICAL VARIABLES (INDEPENDENT VARIABLES) AND THE TWO GROUPS (FORENSIC GROUP VS CONTROL GROUP)**

| **Independent variable (reference category)** | **Odds Ratio [95% CI]** | **p-value** |
| --- | --- | --- |
| **Country of recruitment (Austria)** |  |  |
| *Germany* | 1.20 [0.64; 2.24] | 0.566 |
| *Italy* | 1.07 [0.59; 1.96] | 0.818 |
| *Poland* | 1.33 [0.76; 2.35] | 0.317 |
| *United Kingdom* | 4.45 [1.98; 9.98] | **<0.001** |
| **Sex** (*Female*) | 2.16 [1.21; 3.86] | **0.009** |
| **Children** (*No*) | 2.16 [1.25; 3.73] | **0.006** |
| **Education years** | 0.86 [0.80; 0.92] | **<0.001** |
| **Daily time not engaged** (*0-3 hours*) |  |  |
| *3-6 hours* | 1.42 [0.84; 2.39] | 0.187 |
| *>=6 hours* | 2.63 [1.51; 4.56] | **0.001** |
| **Age of first contact with DMHs** | 1.04 [1.02; 1.07] | **0.001** |
| **Type of SSD diagnosis** (*Schizophrenia*) |  |  |
| *Schizoaffective disorders* | 0.27 [0.14; 0.53] | **<0.001** |
| *Delusional disorder* | 10.30 [1.27; 83.17] | **0.029** |
| *Brief psychotic disorder* | 0.85 [0.05; 13.98] | 0.912 |
| *Schizophreniform disorder* | 4.63 [0.52; 41.14] | 0.169 |
| *Drug-induced psychosis* | 1.97 [0.49; 7.98] | 0.340 |
| **Comorbidity with personality disorders** (*No*) | 4.99 [2.60; 9.58] | **<0.001** |
| **Type of Comorbid personality disorders** (*Other*) |  |  |
| *Borderline personality disorder* | 3.96 [0.34; 46.53] | 0.274 |
| *Antisocial personality disorder* | ND | ND |
| **Collaboration skills in the last year** (*Passively accepts the treatment/intervention*) |  |  |
| *Actively seeks treatment, willing to collaborate* | 0.18 [0.10; 0.34] | **<0.001** |
| *Wants to be helped, but lacks motivation* | 0.29 [0.13; 0.62] | **0.001** |
| *Does not show attention or compreh. for treatment efforts* | 1.70 [0.56; 5.15] | 0.345 |
| *Actively refuses the treatment/intervention* | 0.69 [0.12; 3.97] | 0.678 |
| **PANSS Positive Scale** | 0.97 [0-94; 1.01] | 0.118 |
| **WHODAS 2.0 Total score** | 0.91 [0.88; 0.94] | **<0.001** |
| **BACS – List learning** | 0.98 [0-96; 0.99] | **0.011** |
| **BACS – Verbal fluency** | 0.98 [0-96; 0.99] | **0.007** |
| **BACS – Symbol coding** | 0.96 [0.95; 0.98] | **<0.001** |
| **Frequency of witness of violence** (*Rarely*) |  |  |
| *Occasionally* | 3.03 [0.93; 9.87] | 0.067 |
| *Often* | 4.44 [1.49; 13.26] | **0.008** |
| **Medical attention needed after witnessing violence** (*No*) | 3.85 [1.05; 14.07] | **0.042** |
| **Frequency of victim of violence** (*Rarely*) |  |  |
| *Occasionally* | 1.60 [0.62; 4.11] | 0.327 |
| *Often* | 2.91 [0.95; 8.88] | 0.060 |
| **Beaten, kicked or punched by someone** (*No*) | 2.37 [1.46; 3.84] | **0.001** |
| **When beaten, kicked or punched** (*From early childhood to adolescence*) | 2.80 [1.49; 5.25] | **0.001** |
| **Medical attention when beaten, kicked or punched** (*No*) | 1.96 [0.99; 3.88] | 0.054 |

All ORs are adjusted for country of recruitment and for gender. The OR of country of recruitment has been adjusted only for gender. The OR of gender has been adjusted only for country of recruitment.

ND=Not Defined
